# The Role of Imaging Techniques in Pigmented Bowen Disease and Lentigo Maligna of the Head and Neck: A Comparative Dermoscopic and Reflectance Confocal Microscopy Study

**DOI:** 10.1111/1346-8138.70238

**Published:** 2026-03-27

**Authors:** Federico Venturi, Urszula Fałkowska, Elisabetta Magnaterra, Alberto Gualandi, Carlotta Baraldi, Lidia Rudnicka, Emi Dika

**Affiliations:** ^1^ Department of Medical and Surgical Sciences (DIMEC), Alma Mater Studiorum University of Bologna Bologna Italy; ^2^ Oncologic Dermatology Unit IRCCS Azienda Ospedaliero‐Universitaria di Bologna Bologna Italy; ^3^ Department of Dermatology Medical University of Warsaw Warsaw Poland

**Keywords:** confocal microscopy, dermoscopy, lentigo maligna, pigmented bowen disease, virus related cancers

## Abstract

Pigmented Bowen disease (pBD) may closely mimic lentigo maligna (LM) both clinically and dermoscopically. Accurate differentiation between these entities is clinically crucial, as misclassification may lead to overtreatment or undertreatment. Noninvasive imaging techniques, including dermoscopy and reflectance confocal microscopy (RCM), have improved diagnostic accuracy, but direct comparative data between pBD and LM remain limited. This observational cohort study included 10 consecutive cases of pBD and 10 consecutive cases of LM diagnosed between January and July 2024 at a tertiary referral dermatologic oncology center. All lesions were located on the head and neck and underwent dermoscopic and/or RCM evaluation prior to definitive diagnosis. Epidemiological, clinical, dermoscopic, and confocal features were retrospectively analyzed and compared between groups. The median age at diagnosis did not differ significantly between pBD and LM (64 vs. 56 years; *p* = 0.29). Surface scale was significantly more frequent in pBD (70.0% vs. 10.0%; *p* = 0.020). Dermoscopy showed substantial overlap between groups, although follicular‐centric gray patterns were more commonly observed in LM. RCM demonstrated the highest discriminatory value: disarrayed keratinocytes were present in all pBD lesions compared with 30.0% of LM lesions (*p* = 0.003), while pagetoid cells were significantly more frequent in LM (70.0% vs. 10.0%; *p* = 0.020). In conclusion, a multimodal imaging approach integrating dermoscopy and RCM improves diagnostic discrimination between pBD and LM. RCM provides decisive cellular‐level information and represents a valuable second‐level tool in the evaluation of equivocal pigmented lesions.

## 1 Introduction

Pigmented lesions represent a frequent diagnostic challenge in dermatologic practice, particularly when arising in chronically sun‐exposed areas. Among these, pigmented Bowen disease (pBD), a variant of squamous cell carcinoma in situ characterized by melanin pigmentation, may closely mimic melanocytic neoplasms, most notably lentigo maligna (LM) [1, 2, 3]. Both entities commonly present as slowly enlarging, irregularly pigmented macules or plaques and often share overlapping clinical and dermoscopic features, increasing the risk of misclassification [4]. Accurate distinction between pBD and LM is clinically crucial. LM represents melanoma in situ with a recognized potential for subclinical extension and progression, frequently necessitating staged excision or wider surgical margins. In contrast, pBD is a keratinocytic neoplasm with a different biological behavior and therapeutic approach.

Although HPV infection has been described in Bowen's disease, its presence does not determine the management of pigmented facial lesions. The clinically relevant issue is distinguishing melanoma in situ requiring margin‐controlled excision from keratinocytic carcinoma in situ amenable to conservative treatment.

Diagnostic errors may therefore result in overtreatment, particularly in cosmetically and functionally sensitive areas, or conversely in undertreatment of melanoma in situ [5, 6]. Noninvasive imaging techniques have become central to the evaluation of equivocal pigmented lesions [5, 7, 8, 9, 10, 11, 12, 13]. Dermoscopy enhances the visualization of pigment distribution, vascular patterns and morphology, while reflectance confocal microscopy (RCM) enables in vivo assessment of cellular architecture at near‐histologic resolution. These techniques have individually demonstrated diagnostic utility in both keratinocytic and melanocytic tumors; however, direct comparative data focusing on pBD versus LM remain limited. Moreover, both pBD and LM frequently arise in patients with significant cumulative ultraviolet exposure and a history of keratinocyte carcinomas or melanoma, further complicating clinical assessment. In this context, a systematic evaluation integrating epidemiological data, clinical morphology, dermoscopic patterns, and RCM features is necessary to clarify the respective diagnostic signatures of these lesions. The aim of this study was to compare epidemiological, clinical, dermoscopic, and confocal features of pBD and LM, and to identify imaging features that may reliably aid in their differentiation in routine clinical practice.

**TABLE 1:** Epidemiological, clinical, dermoscopic, and confocal features of patients with pigmented Bowen disease and lentigo maligna.

Clinical features	pBD (*n* = 10)	LM (*n* = 10)	*p* value
Age at diagnosis, years, median (IQR)	64 (59–68)	56 (46–74)	0.29
Male sex, *n* (%)	6 (60.0%)	7 (70.0%)	1.00
History of NMSC, *n* (%)	7 (70.0%)	4 (40.0%)	0.37
History of melanoma, *n* (%)	1 (10.0%)	3 (30.0%)	0.58
Clinical feature			
Ill‐defined borders, *n* (%)	9 (90.0%)	10 (100.0%)	1.00
Multifocal pigmentation, *n* (%)	6 (60.0%)	5 (50.0%)	1.00
Surface scale, *n* (%)	7 (70.0%)	1 (10.0%)	0.020
**Dermoscopic features**			
Pigmented pseudonetwork	6 (60.0%)	4 (40.0%)	0.656
Gray dots/peppering	4 (40.0%)	9 (90.0%)	0.057
Rhomboidal structures	3 (30.0%)	7 (70.0%)	0.179
Asymmetric pigmented follicular openings	6 (60.0%)	6 (60.0%)	1.000
Annular‐granular pattern	4 (40.0%)	8 (80.0%)	0.170
Brown/gray structureless areas	5 (50.0%)	3 (30.0%)	0.650
Scales	7 (70.0%)	1 (10.0%)	**0.020**
Vascular structures	0 (0.0%)	0 (0.0%)	1.000
**RCM features**			
Atypical honeycomb pattern	7 (70.0%)	2 (20.0%)	0.070
Pagetoid cells	1 (10.0%)	7 (70.0%)	0.020
Dendritic cells around follicles	2 (20.0%)	4 (40.0%)	0.628
Disarrayed keratinocytes	10 (100.0%)	3 (30.0%)	**0.003**
Bright nucleated keratinocytes	6 (60.0%)	2 (20.0%)	0.170
Junctional nests	1 (10.0%)	3 (30.0%)	0.582

## 2 Materials and Methods

This was an observational cohort study conducted at the Oncologic Dermatology Unit, IRCCS Azienda Ospedaliero‐Universitaria of Bologna. Cases were selected by identifying 10 consecutive cases of pBD and 10 consecutive cases of LM that were diagnosed and histopathologically confirmed at our institution between January and July 2024. Consecutive case selection was adopted to minimize selection bias and to reflect real‐world clinical practice. All cases corresponded to pigmented lesions located in the head and neck area that were evaluated with noninvasive imaging prior to definitive diagnosis. All included patients had Fitzpatrick skin phototype II or III. Clinical records were reviewed to extract epidemiological and clinical variables, including age at diagnosis, sex, history of non‐melanoma skin cancer (NMSC), history of melanoma, lesion borders (well‐defined vs. ill‐defined), surface characteristics (scaly vs. non‐scaly), and presence of multifocal pigmentation. Images were retrospectively evaluated by experienced dermoscopists blinded to the histopathologic diagnosis. The presence or absence of the following features was recorded: pigmented pseudonetwork, gray dots/peppering, rhomboidal structures, asymmetric pigmented follicular openings, annular‐granular pattern, brown/gray structureless areas, surface scales, and vascular structures [1, 2, 3, 4]. RCM images (VivaScope 3000) were reviewed for epidermal and junctional architecture and cellular morphology. The following RCM features were assessed: atypical honeycomb pattern, pagetoid cells, dendritic cells around follicles, disarrayed keratinocytes, bright nucleated keratinocytes, and junctional nests [10].

### 2.1 Statistical Analysis

Continuous variables were expressed as median and interquartile range (IQR) and compared using the Mann–Whitney *U* test. Categorical variables were expressed as number and percentage and compared using Fisher's exact test. Statistical significance was defined as *p* < 0.05. All analyses were performed using Stata/SE (StataCorp LLC, College Station, TX, USA).

## 3 Results

A total of 20 patients were included, comprising 10 with pBD and 10 with LM. Results are fully displayed in Table 1. The median age at diagnosis was 64 years (IQR 59–68) in the pBD group and 56 years (IQR 46–74) in the LM group, with no statistically significant difference (*p* = 0.29). Male sex predominated in both cohorts (60.0% in pBD vs 70.0% in LM; *p* = 1.00). A history of NMSC was reported in 7 out of 10 (70.0%) pBD patients and 4 out of 10 (40.0%) LM patients (*p* = 0.37). A personal history of melanoma was more frequent in LM (3/10, 30.0%) than in pBD (1/10, 10.0%), although this difference was not statistically significant (*p* = 0.58). Ill‐defined borders were common in both groups, observed in 9 out of 10 (90.0%) pBD lesions and all LM lesions (10/10, 100.0%; *p* = 1.00). Multifocal pigmentation was present in 6 out of 10 (60.0%) pBD lesions and 5 out of 10 (50.0%) LM lesions (*p* = 1.00). Surface scale was significantly more frequent in pBD, occurring in 7 out of 10 (70.0%) cases compared with 1 out of 10 (10.0%) LM lesions (*p* = 0.020).

**FIGURE 1:**
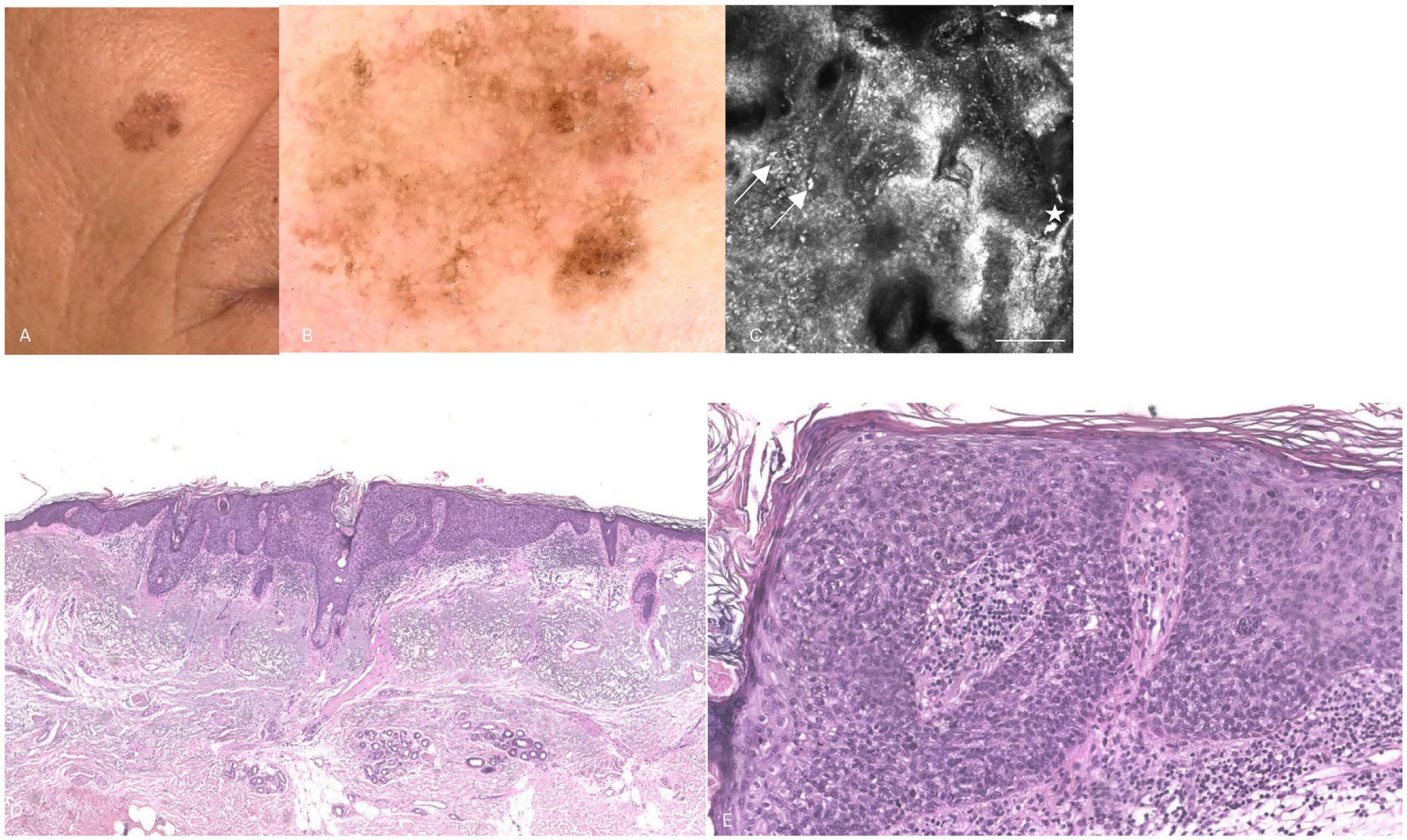
Representative clinical, dermoscopic, reflectance confocal microscopy and histologic images of pigmented Bowen disease: (A) Clinical image showing a pigmented, irregular macule with subtle surface scale and ill‐defined borders on the right cheek of a 59 ‐year‐old female patient. (B) Dermoscopic image revealing scales, brown structureless pigmentation, focal hypopigmentation, and asymmetric follicular openings. (C) Reflectance confocal microscopy image displays bright round and dendritic cells (→) representing pigmented keratinocytes and melanocytes infiltrating the epidermis, and bright round and stellate spots representing melanophages (☆) within the dark dermal papillae. Scale bar = 100 μm. (D) Low‐power view shows full‐thickness epidermal atypia with acanthosis and elongation of rete ridges. The dermis contains a band‐like inflammatory infiltrate (H&E, ×4). (E) Higher magnification demonstrates marked keratinocyte atypia involving the entire epidermal thickness, with nuclear pleomorphism, hyperchromasia, disordered maturation, and scattered mitotic figures. (H&E, ×40)

**FIGURE 2:**
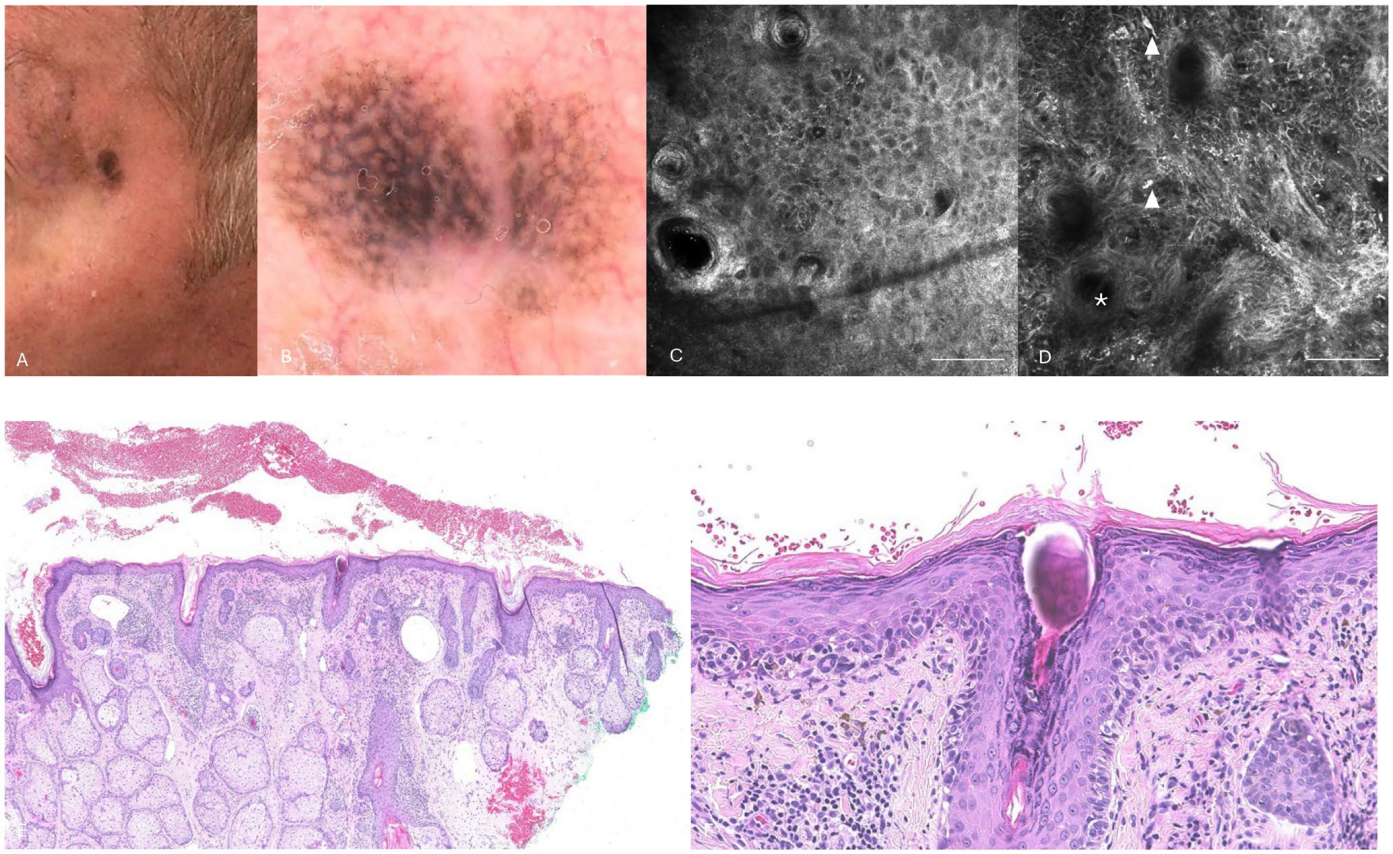
Representative clinical, dermoscopic, reflectance confocal microscopy and histologic images of lentigo maligna. (A) Clinical image showing an ill‐defined pigmented macule with heterogeneous gray‐brown coloration on chronically sun‐exposed skin. (B) Dermoscopic image demonstrating asymmetric pigmented follicular openings, gray dots/peppering, and an annular‐granular pattern. (C) Reflectance confocal microscopy stack from the stratum corneum to the dermo‐epidermal junction layer (D) showing widespread dendritic pagetoid cells and dermo‐epidermal junction disarray (▲) and nonedge papillae (*) at the junction. Scale bars = 100 μm. (E) Low‐power view shows proliferation of atypical melanocytes along the dermal‐epidermal junction, featuring both single cells and nested patterns, particularly involving adnexal structures. (H&E, ×4). (F) Higher magnification demonstrates confluent proliferation of atypical melanocytes along the basal layer with nuclear enlargement, hyperchromasia, and irregular contours. Pagetoid spread is minimal to focal. Atypical melanocytes extend down adnexal structures, a characteristic feature of lentigo maligna (H&E, ×40).

Dermoscopically, pigmented pseudonetwork was observed in 6 out of 10 (60.0%) pBD lesions and 4 out of 10 (40.0%) LM lesions (*p* = 0.656). Gray dots or peppering were more frequent in LM (9/10, 90.0%) than in pBD (4/10, 40.0%), approaching statistical significance (*p* = 0.057). Rhomboidal structures were present in 7 out of 10 (70.0%) LM lesions and 3 out of 10 (30.0%) pBD lesions (*p* = 0.179). Annular‐granular pattern was detected in 8 out of 10 (80.0%) LM lesions and 4 out of 10 (40.0%) pBD lesions (*p* = 0.170). Asymmetric pigmented follicular openings were observed equally in both groups (6/10, 60.0%; *p* = 1.00). Scales were significantly associated with pBD (7/10, 70.0%) compared with LM (1/10, 10.0%; *p* = 0.020). Brown or gray structureless areas were observed in 5/10 (50.0%) pBD and 3/10 (30.0%) LM lesions (*p* = 0.650). No vascular structures were detected in either group (Figures 1A,B and 2A,B).

At confocal observation, an atypical honeycomb pattern was more frequently observed in pBD (7/10, 70.0%) than in LM (2/10, 20.0%), although this difference did not reach statistical significance (*p* = 0.070). Disarrayed keratinocytes were a hallmark of pBD, present in all cases (10/10, 100.0%) compared with 3/10 (30.0%) LM lesions, representing a statistically significant difference (*p* = 0.003). Conversely, pagetoid cells were significantly more frequent in LM (7/10, 70.0%) than in pBD (1/10, 10.0%; *p* = 0.020). Dendritic cells around follicles and junctional nests were more commonly observed in LM but without statistically significant differences. Bright nucleated keratinocytes were more frequently detected in pBD (6/10, 60.0%) than in LM (2/10, 20.0%; *p* = 0.170) (Figures 1C–E and 2C–F).

Surface scale showed 70% sensitivity and 90% specificity for pBD. Disarrayed keratinocytes demonstrated 100% sensitivity and 70% specificity. The combined rule achieved 100% sensitivity with 60% specificity for differentiating pBD from LM. Additional diagnostic performance metrics are provided in the Supporting Information.

## 4 Discussion

pBD and LM represent a diagnostically challenging pair of pigmented skin lesions with overlapping clinical and dermoscopic presentations. In this comparative study, dermoscopy and RCM provided complementary and clinically meaningful information that improved diagnostic discrimination between these entities.

Although biopsy remains the diagnostic standard, a biopsy‐first approach may be limited by sampling error in large or ill‐defined facial lesions and may delay definitive management. RCM provides noninvasive cellular evaluation that increases diagnostic confidence and supports preoperative decision‐making, helping select staged excision for suspected LM while avoiding unnecessarily extensive surgery in pBD.

Simplified imaging criteria showed relevant clinical performance: the dermoscopic scale was highly specific for pBD, whereas disarrayed keratinocytes provided maximal sensitivity. Their combination achieved 100% sensitivity, supporting their use as a practical rule‐out strategy for LM.

RCM can also help with margin assessment in LM by revealing junctional atypia beyond clinically visible pigmentation in selected cases, improving confidence in lesion delineation compared with clinical and dermoscopic evaluation alone and supporting margin‐controlled surgical planning [9, 14, 15].

Further prospective studies in larger cohorts are warranted to validate these imaging criteria and to refine diagnostic algorithms that incorporate noninvasive imaging into routine clinical decision‐making.

## Funding

Finanziato dall'Unione Europea—NextGenerationEU a valere sul Piano Nazionale di Ripresa e Resilienza (PNRR)—Missione 4 Istruzione e ricerca—Componente 2 Dalla ricerca all'impresa—Investimento 1.1, Avviso Prin2022 indetto con DD N. 104 del 2/2/2022, codice proposta: 2022RY8549_002—CUP: J53D23003260006.

## Ethics Statement

The study was conducted in accordance with institutional ethics requirements.

## Consent

Informed consent was obtained from the subject involved in the study.

## Conflicts of Interest

The authors declare no conflicts of interest.

## Supporting information


**Table S1:** Diagnostic performance of simplified imaging criteria for identifying pigmented Bowen disease.

## Data Availability

The data that support the findings of this study are available on request from the corresponding author.
