# Supplementary material for: The Role of Imaging Techniques in Pigmented Bowen Disease and Lentigo Maligna of the Head and Neck: A Comparative Dermoscopic and Reflectance Confocal Microscopy Study
Source: J Dermatol. 2026 Mar 27;53(5):856–60. doi: 10.1111/1346-8138.70238 (PMC13150667; doi:10.1111/1346-8138.70238)
Supplement: Supplementary file 1 — Table S1: Diagnostic performance of simplified imaging criteria for identifying pigmented Bowen disease. [file JDE-53-856-s001.docx]

**Spplementary table 1.** Diagnostic performance of simplified imaging criteria for identifying pigmented Bowen disease.

Sensitivity, specificity, positive predictive value (PPV), and negative predictive value (NPV) were calculated using histopathologic diagnosis as the reference standard. The combined rule was defined as the presence of either dermoscopic surface scale or disarrayed keratinocytes on reflectance confocal microscopy (RCM).

| **Diagnostic criterion** | **Sensitivity (%)** | **Specificity (%)** | **PPV (%)** | **NPV (%)** |
| --- | --- | --- | --- | --- |
| Dermoscopic surface scale | 70.0 | 90.0 | 87.5 | 75.0 |
| RCM disarrayed keratinocytes | 100.0 | 70.0 | 76.9 | 100.0 |
| Combined rule* | 100.0 | 60.0 | 71.4 | 100.0 |

*Combined rule defined as the presence of dermoscopic surface scale **or** disarrayed keratinocytes on RCM.
